# FAST Policing by Telephone: a Randomised Controlled Trial

**DOI:** 10.1007/s41887-022-00083-w

**Published:** 2022-09-06

**Authors:** Stacey Rothwell, Kent McFadzien, Heather Strang, Sumit Kumar, Graham Hooper, Alan Pughsley

**Affiliations:** 1 Kent Police, Kent, UK; 2 Metropolitan Police Service, London, UK; 3 University of Cambridge, Jerry Lee Centre of Experimental Criminology, Cambridge, UK; 4 Cambridge Centre for Evidence-Based Policing, Cambridge, UK

**Keywords:** Differential police response, Tele-policing, Response time, Threats, FAST policing

## Abstract

**Research Question:**

Can caller satisfaction and trust in police be improved (or equalled), after police agree to send a police car to meet with a caller face-to-face, by the alternative of immediate transfer of the call to a police officer who speaks to the caller at length by telephone?

**Data:**

A total of 1016 calls for police service to 999 or 101 assigned by call takers as falling into a “medium” priority category were checked for eligibility, including consent of the caller to speak immediately to a police officer by phone if possible. Eligible offence types excluded domestic abuse but included a variety of other matters. A majority (57.7%) of eligible cases were about threats made by neighbours, workplace colleagues or others known to the caller. A total of 450 cases were selected as eligible for the test sample out of a total of assessed as potentially eligible.

**Methods:**

Eligible cases were randomly assigned to either a *control* group (*N* = 225) of business as usual (BAU) attempts to provide a face-to-face meeting with a police officer, or the *experimental* group (*N* = 225) receiving immediate telephone transfer to a police officer who talked with the caller for over an hour as the initial police response. Analyses were done by intention-to-treat. While 99.75% (*N* = 249/250) of the experimental cases were treated as assigned, only about half of the 225 control cases actually received a face-to-face meeting with a police officer. All 450 assigned callers who gave consent to enter the experiment were contacted for a satisfaction survey at least 14 days following random assignment of the cases, from which the completion rate was 72.5% (almost identical in the two treatment groups).

**Findings:**

Eligible, consenting callers reported substantially higher levels of being “satisfied” or “very satisfied” with the experimental police response by *telephone* (92.6%) than with the BAU efforts to arrange a *face-to-face* meeting between a police officer and the caller (68.9%). Trust and confidence in Kent police declined among 21% of callers receiving BAU service, but only 9% of callers given immediate telephone service. The median time from the initial call to a conversation between police and caller was under 1 min for the experimental treatment vs. 2721 min for the 80% of BAU control treatments in which any conversation between an officer and a caller occurred within 96 h after the call.

**Conclusion:**

This first experiment in a research collaboration on FAST (Finding Alternative and Speedier Tactics) policing has opened the door to further tests of immediate response by remote communications (Rothwell, et al. *Cambridge Journal of Evidence-Based Policing*, *6*, 1–24, 2022). For the kinds of cases included in this experiment, there is a clear preference by callers for the speedier service by a simple phone call over much slower attempts to provide a face-to-face meeting. If broadly adopted across many other high-volume, low-harm categories of requests for police service, fast policing by phone, video or silent live-chat online could improve public approval of policing while allowing more time for police to prevent more serious crimes.

## Introduction: the FAST Policing Collaboration

In early January of 2020, the authors and others convened a meeting in the Kent (UK) Police HQ in Maidstone. The purpose of the meeting was to consider a solution to the dilemma of rising demand for police car responses at a time of reduced numbers of police officers. This meeting became the launchpad of a series of experiments that could change the basic delivery model of police service in liberal democracies. These experiments were retrospectively bundled by Cambridge University partners in the Collaboration with Kent Police as experiments in “FAST Policing”, with FAST chosen as an acronym for Finding Alternative and Speedier Tactics.

At the January meeting, Professor Lawrence Sherman of the Jerry Lee Centre of Experimental Criminology at the University of Cambridge proposed that Kent Police conduct a series of experiments in cutting police time wasted on unnecessary response in person while improving victim experience with police service. He suggested that the benefits of such innovations could include greater investment in preventing seriously harmful violent crime, more proactive policing, and a lower carbon footprint from policing tasks. The series of experiments were calibrated by an escalating level of complexity. Sherman suggested that the first—and simplest—step for FAST policing could be to test an immediate telephone conversation in comparison to a face-to-face meeting with an officer deployed to the caller’s geographic location. The test would be limited to medium risk calls, with careful risk assessment of each caller and call.

In the months that followed that January meeting, the first author of this article designed a master’s thesis protocol for the Cambridge Police Executive Programme, which was supervised by the third author at the Jerry Lee Centre. The successful implementation of that design for the FAST policing by telephone experiment, as reported below, led to further research with more complex technology. On the basis of technical advances in Kent Police capacity for live video communications (developed during a year of intermittent COVID lockdowns), the first author prepared a successful proposal for UK Home Office funding of a second experiment using the GoodSam video communication software for treating cardiac arrest by remote medical guidance (see https://www.goodsamapp.org/). That second experiment in “Rapid Video Response” to calls about suspect-absent domestic abuse cases was reported in this journal in May 2022 (https://link.springer.com/article/10.1007/s41887-022-00075-w), and illustrated in a video posted on YouTube at https://www.youtube.com/watch?v=tGTnZ1wpwqg. It was also presented at the 14^th^ Annual Cambridge International Conference on Evidence-Based Policing, along with the results of the first experiment as reported here [https://www.youtube.com/watch?v=90KnCV2TLqE].

The rapid video response experiment for domestic abuse was highly rated by the victims who received the experimental treatment. They especially praised its speed and the reassurance of seeing a police officer on the screen. The experimental video condition also increased arrests for domestic abuse from 16 to 24% of all calls.

While that second experiment in FAST policing has attracted a substantial readership and interest from over 30 other police agencies in the UK and abroad, it would not have happened without the first test done by telephone, without any video capacity. That initial test is what the present article reports. The use of simple telephone technology offers an even wider scope for FAST policing than rapid video response, if only because there are many more telephones *without* internet video communications that could be used to contact police. In India, for example, there were “1.2 billion mobile subscribers in 2021, of which about 750 million [were] smartphone users” (https://www.business-standard.com/article/current-affairs/india-to-have-1-billion-smartphone-users-by-2026-deloitte-report-122022200996_1.html). That still left 450 million people unable to access police by video who could still call the police by voice-only mobile phones. In the UK, by contrast, an estimated 92% of all mobile phones were smartphones, with over 85% of the population using smartphones (https://cybercrew.uk/blog/how-many-people-own-a-smartphone-in-the-uk/). Yet, this also means that millions of people could not contact police in an emergency by using video, even though they can call police by voice-only telephone.

The contrast between the present test and the video experiment is not only about the medium of communication. More important, perhaps, is that the video experiment was limited to domestic abuse cases, while the first experiment excluded them. From an offence-category perspective, it is useful to place the current report in the category of testing FAST policing for non-domestic cases, and the previously published video experiment (Rothwell et al, 2022) in the category of offender-absent domestic abuse. This leaves many other categories of offences leading to calls for police assistance that should be tested for the safety and effectiveness of FAST policing. Given the important question of whether routine (but always by caller consent) use of alternative speedier tactics may increase harm to the callers, there is a strong case for launching all tests of new categories as randomized controlled trials. Building such a strong base of evidence would not only help to protect the safety of the callers. It would also help to protect the legitimacy of police decisions to offer FAST service in the event of an extremely harmful outcome, such as death of a caller that might have been just as likely to occur under BAU response.

### UK Context: 2020

As of January 2020, UK police faced the risk that demand would overwhelm capacity (Her Majesty’s Inspectorate of Constabularies and Fire Response Services (HMICFRS), 2020). Calls for service in England had nearly trebled in the past two decades, with police forces during 2018 receiving over 26 million calls (Policing Insight, 2020). Emergency 999 calls to police forces increased by 11% between 2016/2017 and 2018/2019 (HMICFRS, 2020).

Police forces’ ability to meet and manage demand was being tested more than ever before. The UK saw the largest population growth in 70 years whilst experiencing a decade of police budget cuts. The cuts of police officer numbers by a third (APD Communications, n.d.) led to call numbers far outstripping capacity to respond (Walley and Jennison-Phillips, 2020; Dunnett et al., 2019; Laufs et al., 2020; Waddington, 1999).

The House of Commons’ Home Affairs Select Committee in 2018 acknowledged that the police are “struggling to cope” as a result of the changing type and rising number of crimes, combined with a decline in police numbers, outdated technology and structures (Home Office Affairs Committee, 2020). There was every expectation that demand and complexity will continue to rise (HMICFRS, 2020; NPCC, 2019; Laufs et al., 2020).

Kent Police have been no exception to these trends. As a medium-sized English county police force covering an area of 1443 square miles, Kent had a population of some 1.8 million people. They were served by 3780 serving police officers and 2675 police civilian employees at 31 March 2020 (Home Office 2020). The Force Control Room (FCR) remains the hub of the organisation, receiving and responding to all calls for service. Call Takers are responsible for receiving and triaging emergency (999) and non-emergency (101) calls for service, and dispatchers are responsible for the prioritisation and resourcing of them, dispatching and communicating with frontline police officers.

During 2019, there was a total of 807,650 telephone calls received by Kent Police Call Takers. From these calls, 271,124 incidents were created; 195,722 of them were assessed as requiring physical attendance by a police officer. This amounted to a mean of 536 incidents that required servicing every day, solely reported by telephone calls from the public.

Deluged with demand, control rooms have the unenviable task of trying to match the calls for help to an appropriate, proportionate and more importantly, available response (Lum et al., 2020; Waddington, 1993). The levels of demand are unpredictable and unlimitable. But the response taps a finite resource—police officers and staff—and a finite variety of response options that are rarely based upon evidence (Sherman, 2013), that have not changed for forty years. These options are as follows:

- Officer in-person response either immediately or in a delayed manner
- Scheduling of the call for a later appointment
- The call to be dealt with administratively over the telephone by the call taker (resolved without deployment (RWD))

This triage model of response is no longer adequate, with calls prioritised, stacked and delayed (Lum et al., 2020, Waddington, 1993; HMICFRS, 2020; Ekblom & Heal, 1982; McEwen et al., 1986). If and when a patrol eventually arrives, its response will often be hurried and unsatisfactory for the complainant (Antunes & Scott, 1981).

The impact of this demand is felt across the force with a range of administrative functions and triage processes involving constant re-assessment and re-prioritisation as they are not being serviced as quickly as they are being populated. The far too frequent outcome is a delay for the victim or no attendance at all. With demand and complexity of calls continuing to rise, strategic leaders of Kent Police decided to explore a radical and different approach to servicing calls, initially known as the Rapid–Telecommunication Response to Emergency (and other) Calls (R-TREC), and later to be enfolded in the portfolio of FAST Policing experiments as described above.

R-TREC was an immediate telephone response to a call for service by a warranted police officer (equivalent to a “sworn” officer in US terminology). It was offered as an alternative to a non-urgent face-to-face in-person response by a warranted police officer. The decision was to test this innovation with a randomised controlled trial (RCT), the results of which are reported here.

## Research Question

*Can caller satisfaction from agreeing to send a police car to meet with a caller face-to-face be equalled (or exceeded) by the alternative of immediate transfer of the call to a police officer who speaks to the caller at length by phone?*

## Data

### Summary of Data Collection

Two strands of data—one concerning the differential deployment of resources and the other concerning victim satisfaction—were collected and analysed. Both were limited to a carefully selected subset of all incoming calls.

For the purposes of this study, relevant eligible calls were defined as those of a non-urgent nature that normally required physical attendance (in Kent, described as “priority” calls, as distinct from “immediate” calls needing urgent despatch). They were identified within the FCR as they were in-coming and the line not yet been cleared by the victim.

Once the victim confirmed that they were willing to receive the R-TREC treatment (if available), they were randomised equally into two groups. The treatment group received the R-TREC service and were immediately transferred to a waiting police officer, who provided the response service via the telephone. The control group calls were placed on the dispatcher’s list as per business as usual (BAU) and awaited a non-urgent physical response.

Following completion of FCR call-handling, all callers who consented to participate in the study were called by a telephone survey team. The survey of those who responded to survey calls asked questions about the call and their level of satisfaction with how police responded to the call.

### Records of Call Processing in the Force Control Room

In Kent, police call takers receive telephone calls for service and initially create a unique record using the computer-aided dispatch (CAD) system. The call takers determine priority with a call grading, assign the call type and record personal and incident details, and inform the victim of the likely service they will receive. The CAD record is then passed electronically to a dispatcher who will prioritise the CAD according to its grading. The Dispatcher has oversight of all the available patrols, controls the radio communication with the officers and dispatches officers to the calls, when relevant and available.

Nationally in England, emergency calls are graded as “immediate” when they present a danger to life, the use or immediate threat of use of violence, serious injury to a person or serious damage to property. Police officers respond to “immediate” calls as quickly as possible and usually in a vehicle with lights and sirens. None of those calls were eligible for inclusion in this study. The calls eligible for this study were categorised as *priority*, i.e. there is a degree of seriousness requiring deployment to the incident as soon as possible. Historically there were national guidelines around attendance times for these calls, but now there is no set period (HMICFRS, 2020). Attendance for these priority calls does not happen in all cases and when it does, there can be a substantial delay depending upon call demand and officer availability.

In 2019, Kent Police created 271,124 incidents or CADs. These incidents were graded by call takers to establish the type of response that would be given. They graded 94,066 incidents as requiring a priority, non-urgent and in-person response, of which 49,509 were judged to be eligible for this trial. Officers were dispatched to 52,096 (55.4%) of these incidents. The mean time it took to arrive at an incident was 20 h and 14 min, of which the mean journey time for those that received attendance was 24 min.

### Two Time Periods: a COVID Requirement

The R-TREC randomised controlled trial was conducted across two time periods due to the COVID-19 pandemic. Phase 1 ran for 10 days in March 2020 (95 cases) and phase 2 ran for 50 days between July and September 2020 (355 cases), for a total of 450 cases with 225 randomly assigned to each condition. This break in the study meant that the trial was conducted in two phases, in a block RCT design so that the results could be analysed both separately and combined, before and during COVID-19 restrictions. The hours of operation varied during the trial, but core hours were late morning and afternoons. The trial generally operated with two or three officers taking the calls with the first author having continual oversight of the incoming calls. Analysis of the distribution of sex, age, disability and ethnicity of the victims revealed no significant differences between the treatment and control groups.

## Methods

The R-TREC treatment required the immediate transfer of the call from the FCR call taker through to a police officer to provide the first response service via telephone. Traditional call transferring technology was used to move the call, *with the caller still on the line*, from the call taker, through the Research Team and onto the R-TREC officer. The process would routinely take less than a minute and was a reliable method of transfer. This capability ensured that there was no loss of contact with the caller and that the response could be considered immediate. The R-TREC officer would provide the first response to the call, immediately engaging with the victim, often taking details of the crime, undertaking immediate safeguarding, immediate referrals to partner agencies and securing evidence.

The R-TREC officers were not told how they were to undertake their duties, other than to say that they should provide the first response service to the incident as if they were in attendance.

The control group calls were given the BAU response and placed on the dispatcher’s list, with the ambition of responding with non-urgent physical attendance, though they might ultimately receive an alternative response, including (1) non-urgent attendance (some days later), (2) a scheduled appointment at the victim’s convenience or (3) agreeing that the call was resolved without deployment of a police officer.

### Eligibility Criteria

The eligibility criteria for inclusion on the study were as follows:

1. The call had been graded as priority
2. Was not domestic abuse (DA)
3. Was still live on the line
4. The caller had opted into accepting a telephone service if available
  In addition, *exclusion criteria* were applied through *risk assessment:*
5. If prompt physical attendance was *necessary* to prevent injury, protect property, pursue offenders or prevent further crime

If there was no officer available to provide R-TREC, the case was recorded, given the BAU response and excluded from the trial. Once eligibility had been finalised, the case was randomly assigned via the Cambridge Randomiser (Linton & Ariel, 2020).

Following checks for eligibility, and the exclusion of 566 cases after risk assessment, a total of 450 cases were randomly assigned across the two study periods, 225 to each condition. With no significant differences between the participants in the two periods, combined results are presented here. All analysis is based on “intention to treat” protocols.

**Fig. 1:**
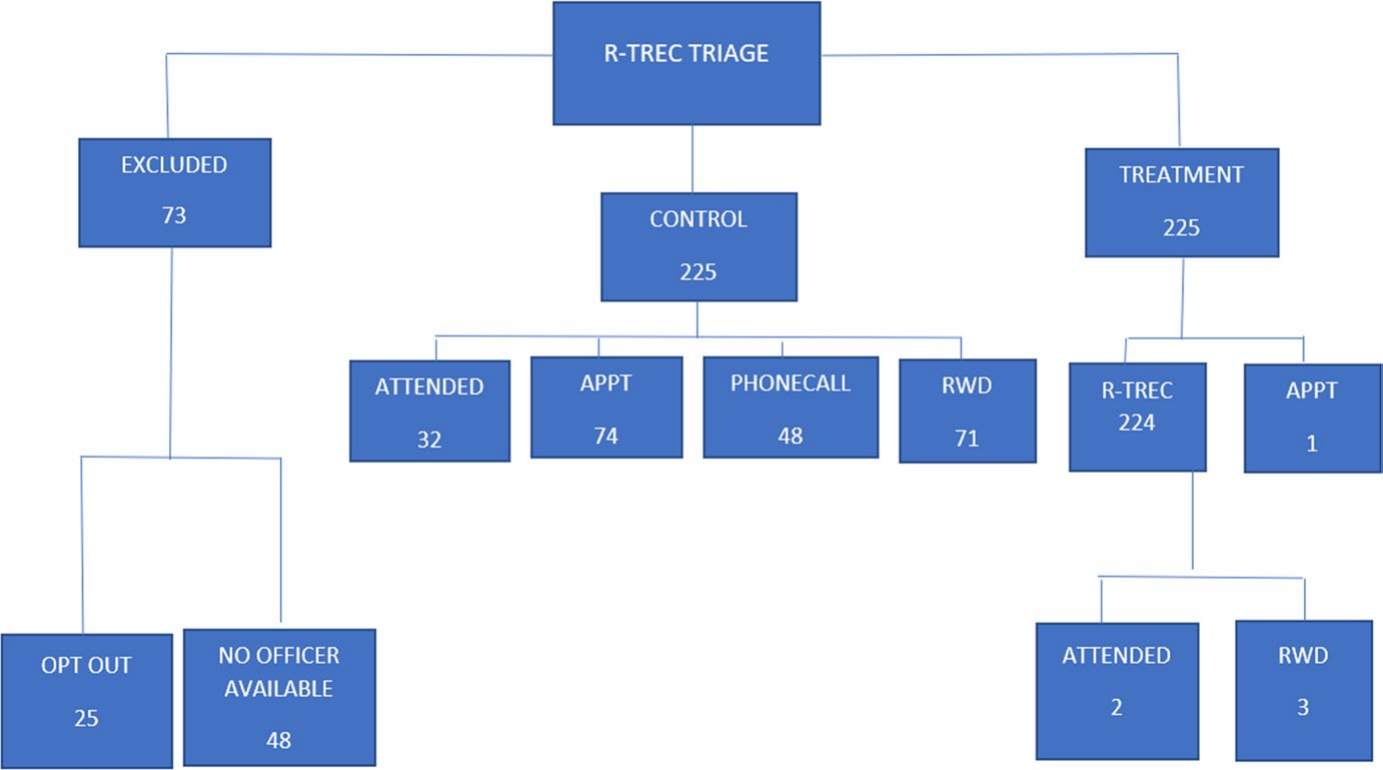
Case flow through the study

All but one of the 225 cases randomly assigned to the experimental phone officer treatment received treatment as assigned (referral to a phone officer, with whom the caller spoke). In five of the referred cases, the phone officer decided to dispatch a car immediately. The 225 in the control condition all received BAU, with four possible dispositions of the BAU cases, as displayed in Fig. 1.

### Nature of the Caller’s Problem: 21 Incident Types

**Fig. 2:**
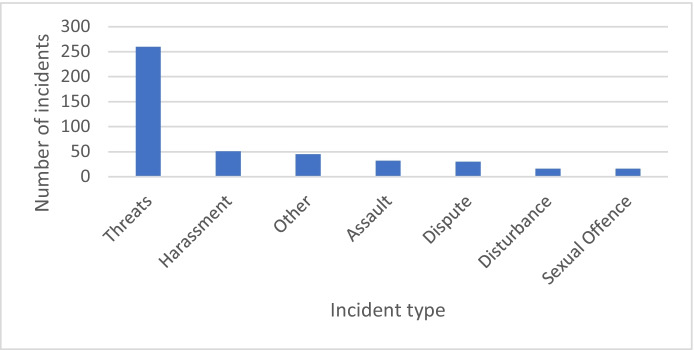
Most frequently occurring incident types that were included in the study

The study included 21 different types of incidents. The largest of the incident types was threats with 260 (57.7%) of the 450 eligible incidents falling into this category. Other categories with at least 3.5% or higher were sexual offences, disputes, assaults and harassment (Fig. 2).

**Fig. 3:**
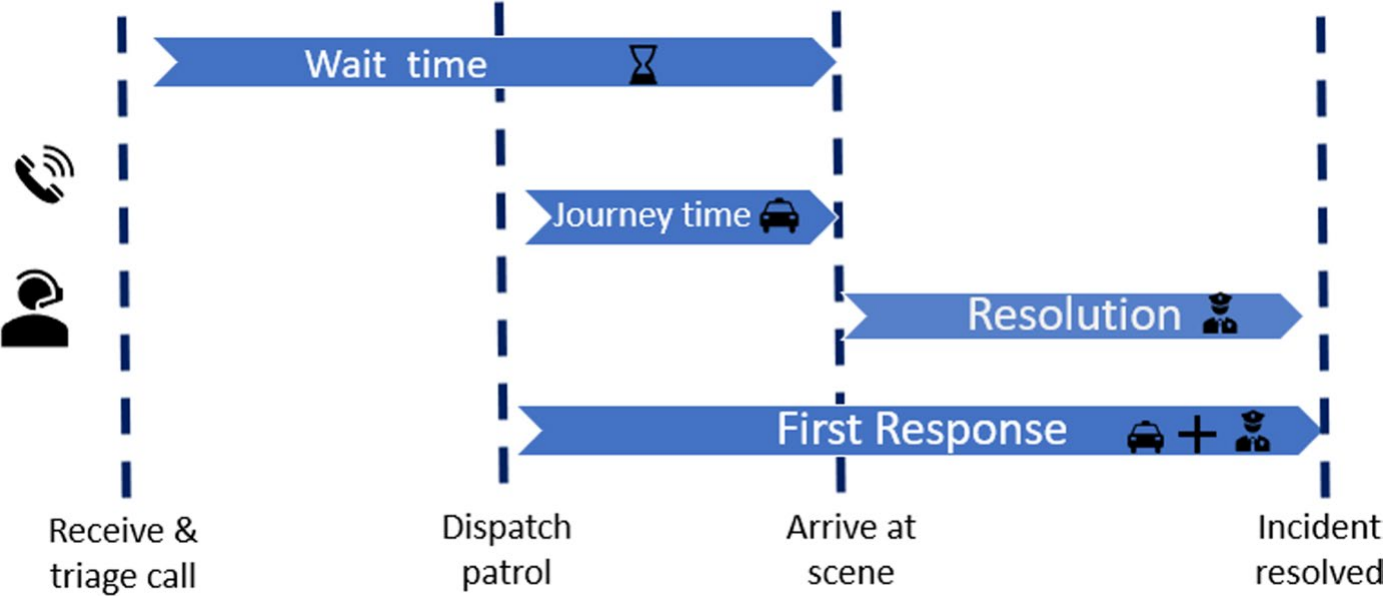
Measurements of time for successive phases of response

Outcomes focused on two measures. One was police officer efficiency, which was determined by measuring and comparing time periods in the two conditions (Fig. 3).

The second measure, derived from telephone survey research, compared levels of victim satisfaction in the two conditions. This outcome was of primary concern to Kent Police because victims’ expectations of how their call for service is handled directly affects their views of police legitimacy (Bartsch & Cheurprakobkit, 2004; Dean, 1980).

A telephone-administered victim survey was designed to capture the views of both the treatment and control group victims. The survey consisted of twelve questions that aimed to capture overall satisfaction, expectations about timeliness and the effect of any delay, levels of confidence and trust, views on telephone response and whether they would recommend R-TREC in the future. Victims were contacted at least 2 weeks after the date of the call for service, to ensure they had received the first response.

## Findings

### Service to Victims

#### Wait Time

Temporal measures of victim service show that for “wait time” (i.e. the time the victim waited for a live response by a police officer), the median for the treatment group was 0 for 249 of the 250 incidents (one case was misassigned). For the control group, the median was 1 day, 21 h and 20 min. Assigning an arbitrary benchmark of 1 min to round up from the less than 1 min wait time for treatment cases, the 45 h and 20 min wait equalled 2720 min. Put another way, the FAST policing by telephone cut the wait time by over 99%, for a service that was 2700 times faster.

#### Resolution Time

Resolution time is the time a police officer actually spends with the victim, whether on the telephone or in person. On this measure, there was essentially no difference: a small but statistically insignificant difference between the mean of 78 min for the treatment cases and 73 for control cases (t = 1.09, p = 0.27).

**Fig. 4:**
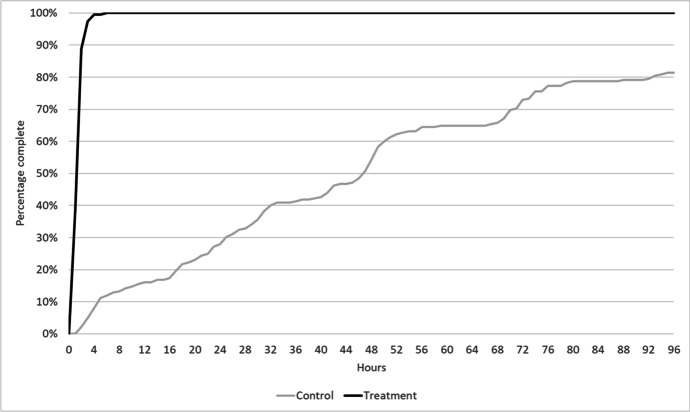
Wait time timeline for all incidents receiving a completed response (disaggregated)

Figure 4 shows a timeline for when the treatment and control groups received a first response. While 100% of the experimental cases were completed within 4 h, almost 20% of the BAU cases remained unresolved 96 h after the call was received.

### Victim Satisfaction

Telephone interviews with victims were conducted over several weeks beginning 2 weeks after the call for service and achieved an overall response rate of 72.5%, almost identical in each condition.

**Fig. 5:**
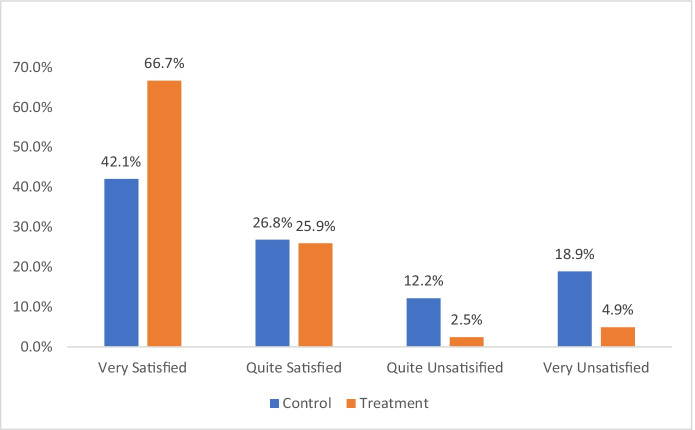
Victim satisfaction

Figure 5 shows how satisfied victims were with the way the police officer dealt with their incident. While 68.9% of BAU (control group) callers were satisfied or very satisfied, 92.6% of the experimental group callers were satisfied or very satisfied. In the “very satisfied” category, FAST policing by phone attracted 34% higher ratings than BAU. Conversely, the callers receiving BAU were almost 4 times more likely (3.85) to say they were very unsatisfied with police response than those receiving immediate police service by phone.

**Fig. 6:**
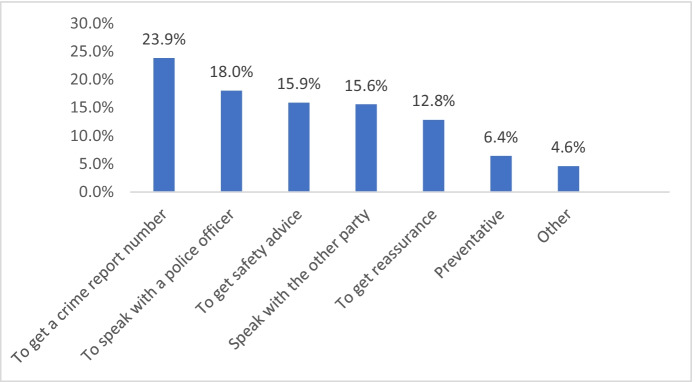
Victims’ reasons for calling the police

Figure 6 shows the main reasons victims gave for calling the police. These reasons generally suggest that what callers required consisted largely of conversation with police, rather than a desire for police to take any action at the location from which the caller was calling.

**Fig. 7:**
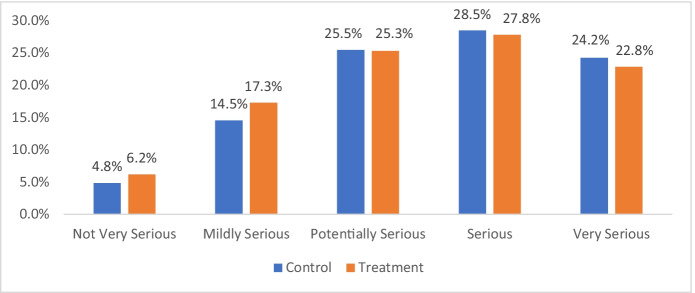
Victims’ view of the seriousness of the incident

When victims were asked how seriously they graded the incident they called about, Fig. 7 shows that over half of them saw it as serious or very serious.

**Fig. 8:**
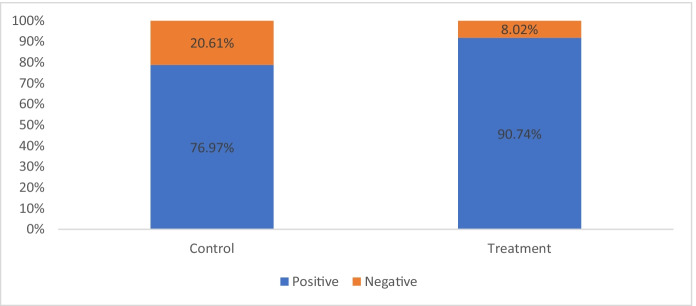
Rating of the manner with which police responded

When victims were asked how they would rate the manner in which police responded, Treatment group victims rated the service more positively than those in the Control group. There is a statistically difference in satisfaction between them (*x*^2^ = 10.84, *p* < 0.01) with an effect size rated small to medium (*d* = 0.48) and a relative percentage difference of 18% higher frequency of positive ratings for the phone service than deployment of a police car (Fig. 8).

**Fig. 9:**
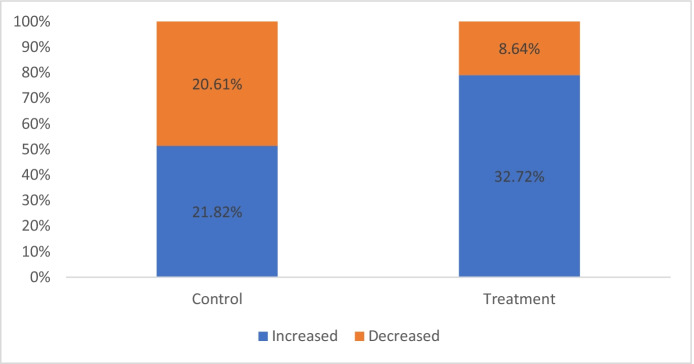
Victims’ level of confidence in Kent Police

Victims were asked whether their confidence in the police had changed as a result of the way police responded to their call. About half of them said their view had changed. Figure 9 shows the direction of change for these victims. There is a statistically significant difference between the groups (*x*^2^ = 11.52, *p* < 0.01) with an effect size rated medium to large (*d* = 0.61) favouring the treatment group. While over 1 in 5 callers receiving BAU said their trust in police had declined, fewer than 1 in 10 callers receiving immediate phone service reported a decline in trust. Almost identical results were found when victims were asked about changes in their trust in the police.

When treatment group victims were asked whether they would recommend R-TREC to others in similar circumstances, almost 90% of them said they would do so.

### Police Efficiency

While the victim’s perspective remains the primary consideration in evaluating the success of R-TREC, police efficiency and costs of delivery are also important criteria. Here, we examine differences in journey time, first response time and resolution time between the treatment and control groups and calculate the number of “officer minutes” each job received for cost/benefit purposes.

Journey Time. Only 84 (37.3%) of the 225 control cases actually received physical attendance at the victim’s address and incurred journey time. Table 1 shows journey times for both control and treatment cases receiving a physical visit, with the single treatment case constituting a violation of the experimental protocol.

**Table 1:** Journey time

Journey time	Received a physical visit	Mean	Median	Maximum
Control	84	16.74 min	14 min	45 min
Treatment	1	0.14 min	0 min	32 min

#### First Response Time

Table 2 shows a statistically significant difference in mean first response time between control and treatment (*t* =  − 3.42, *p* < 0.01), with a small to medium effect (*d* =  − 0.32) of more police time consumed by BAU than by the telephone service.

**Table 2:** First response time

First response—efficiency (journey + officer time-all attending officers)	Mean	Median	Maximum	Minimum
Control-deployed (*n* = 154)	2 h 5 min	1 h 35 min	10 h 37 min	15 min
Control-resolved without deployment (RWD) (*n* = 71)	51 min	31 min	5 h 32 min	0 min
Control (*n* = 225)	1 h 42 min	1 h 15 min	10 h 37 min	0 min
Treatment (*n* = 225)	1 h 18 min	1 h 8 min	5 h 32 min	17 min

#### Resolution Time

When comparing resolution time, control cases took on average 14 min longer than treatment cases, a statistically significant difference (*t* =  − 2.19, *p* < 0.03) (Table 3).

**Table 3:** Resolution time

Resolution time (total minutes of all attending officers)	Mean	Median	Maximum	Minimum
Control (deployed) (*n* = 154)	1 h 52 min	1 h 23 min	10 h 24 min	0 min
Control (RWD) (*n* = 71)	51 min	31 min	5 h 32 min	0 min
Control (*n* = 225)	1 h 32 min	1 h 6 min	10 h 24 min	0 min
Treatment (*n* = 225)	1 h 18 min	1 h 8 min	5 h 52 min	17 min

#### Officer Minutes

All the treatment cases received a response from only one officer at a time. Forty-nine of the 225 control cases received two officers at a time, and one case received three officers, while 173 received only one officer at a time. Table 4 shows the average number of officer minutes spent per job and the calculated costs per job based on the hourly cost to Kent Police of a probationer constable in 2021 (£31.46). The cost per call has been calculated by multiplying the cost per minute of an officer (£0.524) by the mean number of minutes per call.

**Table 4:** Cost of Incidents serviced

	First response-officer minutes per call (mean)	Cost per call
Treatment	1 h 18 min	£40.90
Control	1 h 42 min	£53.48
Control-attended	2 h 33 min	£80.22

Of the total incoming incidents during the operational hours of the trial, about half were eligible after applying risk assessment. In 2019, there were almost 50,000 calls to Kent Police that fitted the incident type criteria used in the trial. The study team estimated that close to 24,000 of these—almost half—could have been dealt with by R-TREC. Table 5 shows a conservative estimate of the savings for Kent Police using the saving difference between the control and treatment cost per call calculation (£53.48 − £40.90 = £12.58).

**Table 5:** Potential resource savings from R-TREC

	Resource savings per incident	Number of calls	Annual resource savings
R-TREC	£12.58	23,764	£298,951.12

There are vehicle and fuel savings as well in respect of those incidents in the Control group who were physically attended (84 of the 225). Kent Police estimate cost per mile at £1.33; the average miles travelled per attended incident was 4.63 for the control group, so the average cost per journey was £6.16 and total savings for the control group were £517.16. Table 6 shoes the estimated annual transport savings if the same proportion of calls (37%) were responded to and travelled as in the trial.

**Table 6:** Annual transport savings

	Number of calls potentially travelled to	Vehicle and fuel cost per call	Potential annual vehicle and fuel savings
R-TREC	8793	£6.16	£54,164.88

## Discussion

This study aimed to discover the effects on victim satisfaction and police costs of diverting intended visits to an immediate phone service with a warranted police officer. The results from this RCT clearly demonstrate both higher victim satisfaction and lower costs associated with the immediate phone service response, R-TREC.

To understand the policy implications of these findings, we must understand the level of demand that Kent Police, like others across the country, are currently facing. Growing demand detracts from the ability to provide the intended response, or in some cases to provide an officer response at all.

The intended in-person response to the control group entailed lengthy wait times and a large but inconsistent array of response services. Only a small proportion (14%) of the cohort received the intended service of a non-urgent physical attendance. Most receiving the standard treatment (without immediate phone service) were provided an alternative resolution after a lengthy delay..

In contrast, the R-TREC treatment was almost always delivered as promised. That promise was immediate service, to all but one of the 225 cases in the study. Additional attendance was only required in two cases incurring resource cost, both of which facilitated the early apprehension of an offender and the ability to protect a vulnerable individual which otherwise may not have happened.

The efficiency findings of the study show that there are savings to be had with R-TREC, even though an officer in-person response was not delivered to one-third of the control group. Savings would increase substantially if one were to deliver the intended in-person response to every priority-graded call.

Victim satisfaction overall was substantially higher for R-TREC than for BAU: 92.6% compared to 68.9%. Nine out of ten victims interviewed would recommend R-TREC. This result is in line with that of McEwen et al. (1986), whose research in three US cities found that less costly alternative response options could be offered whilst maintaining victim satisfaction. These results are also consistent with those of Ekblom and Heal (1982) in the UK, showing that differential police services could be offered whilst maintaining victim satisfaction.

The victim survey showed that the response delays affected the victim’s perceptions of legitimacy in the control group, resulting in a decrease in confidence and trust in police. With the impact of a negative experience having up to 14 times greater impact than that of a positive one, this brings into focus the importance of victim satisfaction when improving response services (Skogan, 2006).

There was also a substantial difference in wait time, with control group victims waiting on average 2 days, 10 h and 5 min for a response compared with the near-immediate response of treatment of 14 min for the treatment group. There was less anxiety about timeliness of response within the experimental group, with no callbacks to police and several victims commenting on the speed of response.

In contrast, victims from the control group expressed their disappointment in the delay and that the service had not met expectations, with comments such as “I thought someone would have come sooner”. and “I thought it would be quicker than it was as I was scared”. Almost half of the control group (46.7%) said that they expected police to come straight away, soon or that same day.

Service failure was said to have had a significant impact on 35.2% of the control group victims, and their dissatisfaction was evident in some of their comments: “We were expecting someone to come and they didn’t. I sat and waited for police and no one came.” and “We waited 36 h and they didn’t come”.

Over half of the control group (52.7%) described the incident that they were reporting as serious or very serious. The consequences of service failure for a small number of victims were significant; These findings support the claims made by Laufs et al. (2020), the Home Office Communication Directorate (2005) and the NPCC (2019) that unmanageable levels of demand lead to victim dissatisfaction and decreases in perceptions of legitimacy, and in confidence and trust, in the police.

In contrast, the service given to the R-TREC treatment group was well received, with nine out of ten callers receiving that service (90.7%) rating the manner with which the police responded positively. An important benefit of an immediate response service is that officers have oversight of an incident instantly, providing reassurance and direction to a distressed victim at the point of call. As the callers noticed, R-TREC succeeded in removing the three or four steps in the service normally involved when calling for help (Walley & Adams, 2019).

Mobile phones and improved phone signal and Internet connectivity now allow calls for service to be made soon after a crime has been committed or is discovered, when victims are in a heightened state of distress (NPCC, 2019; Home Office Directorate, 2005). The immediate relief that an R-TREC officer can provide was palpable with one victim who said: “I was really shocked that I spoke to a P.C. I felt YES! This is quite serious, and they are listening to me…That helped with my confidence in Kent Police…because it went straight to a police officer”. Officers within the treatment group were able to provide important safeguarding advice and fast-time referrals to partner agencies to protect victims from further harm. Meanwhile, victims in the control group awaiting a response could not be afforded such protection, so situations had the potential to escalate—creating further harm and even more demand.

The difference between the control and treatment response service was not just the speed of delivery but also the type and consistency of interaction. The control group received an array of methods of communication: in-person, via telephone, scheduled and response without deployment. Officers within the treatment group were able to devote time and attention to the victim and were not distracted by their personal radios or diverted to a more urgent call, enabling a quality service to be delivered (Gay et al, 1977; Sumrall et al, 1981).

There are some additional benefits for the victim receiving a telephone response. These include the ability to service a call discreetly and at a convenient time for the victim comfortable in their surroundings, and without neighbours’ prying eyes. Victims in the trial were asked if they would consider other remote service offerings, specifically video. Three-quarters of the entire cohort said that they would choose a video response in the future, supporting the claim made by the NPCC (2019) that having issues resolved remotely in other parts of their lives means that victims are comfortable with and hold similar expectations for police resolution services.

This study cannot determine whether the increase in victim satisfaction in the Treatment group was caused by the fast response, or expectations being met, or the optional offering, or the method of communication used, or ultimately a combination of all of them. What can be concluded is that the treatment response gave a much higher rating of satisfaction than the control and has been shown to be more effective in delivering what victims want.

The effectiveness of a new police service such as R-TREC in delivering what victims want must be weighed against the economic efficiency of the intervention to ensure public value. The main efficiency savings were achieved by finding alternative, speedier tactics (FAST) to sending two officers for an in-person visit, as well as the travel time and associated costs for engaging in essentially the same kind of conversation. There was only a small difference between the treatment and control group’s resolution time: officers in the treatment group spent on average 5 min longer with the victim and conducting enquiries than in control cases.

Overall, R-TREC cost half that of an incident physically attended and was one fifth cheaper than the overall aggregated BAU response. These cost savings exclude calls graded for a scheduled appointment, as well as calls received via other methods such as online calls for service and the wider call types that were excluded from the trial. Further efficiency gains can be realised from using officers working remotely, making the best use of officers who cannot serve on the front line for medical or health reasons. Special Constables could also be utilised. There would, however, be equipment cost implications for R-TREC that have not been included within these calculations, for the purchase of any laptop or headset for the officer when working remotely. Furthermore, the R-TREC officers would need to be utilised for other administrative tasks whilst waiting for incoming calls, and this would need to be factored into any decisions regarding resource numbers.

Outside of the parameters of this study, there are additional ancillary time and cost savings to be considered. These include.

- Eliminating the need to re-read and re-assess incidents on the dispatcher’s open lists
- The costs of attempting to deploy to an incident that is then subsequently diverted due to an emergency call
- The cost of sending an officer to meet a caller who is not at the address when police arrive
- The financial and reputational cost of investigating complaints regarding lack of or timeliness of response

## Conclusions and Policy Implications

The most significant policy implication of this experiment is that victims greatly favour R-TREC over waiting for police to arrive in person. With 95% of callers opting to use the R-TREC service during the study, it is important to recall that three quarters of all victims (78.2% of the control group and 75.9% of the treatment group) deemed their incident as either potentially or actually serious or very serious (Fig. 7). Given that the public recall bad experiences with the police far more than good ones (Skogan 2006), these policy implications are most relevant to police leaders, whose duty is to provide police services efficiently and effectively.

Another policy implication to consider is one felt by the frontline. Previous studies have shown that Call Takers are risk-averse and tend to “think car”, meaning that they start on the presumption that officers need to attend (Waddington, 1993, 1999). This behaviour overloads the dispatch lists. By reducing the list with a FAST option of R-TREC, call takers and dispatchers can better manage the constant supply of calls for service. Call takers’ judgment decisions would no longer dictate what is on the list, taking the responsibility away from the call taker and giving it to the officer (Kleinig, 1996).

Triage would then only be required to assess suitability for the response option, eliminating the need for unwieldy, complex, repeated risk assessments. BAU relies on subjective decision-making and prioritisation of calls in control rooms. It is difficult to establish whether these judgement decisions are made correctly (Kleinig, 1996; Laufs et al., 2020). R-TREC officers, with the assets of both more time and experience than most call takers, can help to protect both the victim and the organisation against any mistakes made during the swift triage and grading process within an emergency control room.

Meanwhile, officers and FCR staff can devote their time and attention to prioritising incidents, and dispatching patrol cars to attend those incidents that demand physical attendance (Gay et al., 1977; Sumrall et al, 1981). R-TREC removes the need to prioritise entirely by redefining the frontline. This in turn has a subsequent positive benefit for the FCR.

The findings of this experiment suggest that reducing the delay in engaging with the victim and improving the speed with which the crime is recorded and investigated may have many positive implications. The (UK) College of Policing’s “Golden Hour” principles promote expediting crime recording and the fast time collection of evidence (C.O.P, 2013). R-TREC officers were able to undertake immediate intelligence checks, assign immediate taskings for both forensic examination and CCTV collection, and even obtain witness statements at the point of call. Future research may determine whether R-TREC improves outcomes further along its investigative journey, such as greater likelihood of victims supporting prosecutions, reduced repeat victimisation, or reduction in crime harm. While all of these potential benefits are beyond the remit of this study, future research could explore them with the same kind of randomised trial design.

Finally, we must be aware of several limitations of the study design. One is the possibility that the control group’s lower satisfaction may have been affected by the fact that they were not able to have the R-TREC service that they had said they were prepared to accept (as an eligibility criterion for inclusion in the study). Another is that the Hawthorne effect of the research team’s enthusiasm may have had an impact on the R-TREC officers who were aware of the study, unlike the officers that provided a response in the control group (Wickstrom and Bendix, 2000). Third, R-TREC officers (but not control case officers) were aware that victim interviews were going to be conducted for the study, though it is common knowledge to all officers that Kent Police regularly undertake satisfaction surveys with all kinds of victims.

Fourth, it should be borne in mind that R-TREC was tested only on selected call types and call gradings and only during certain times and days of the week, due to operational constraints. A number of calls were excluded by the study’s dispatcher, who applied a subjective risk assessment which was subject to human error, human decision making and possible selection bias.

The evidence emerging from this study suggests that FAST policing by telephone should be considered more broadly across the range of policing responses. Immediate telephone (or video) response could improve police performance not only for “priority”-graded calls (as in the present study) but also those that are lower in risk, such as scheduled appointments. Immediate conversations could also be tested for use in “immediate”-graded incidents, the highest grading of emergency calls. New experiments may reveal opportunities for early engagement with victims, the ability to command major incidents, prevent injury, capture evidence and identify offenders, all whilst awaiting officer arrival in potential life or death situations.

Police control rooms use sophisticated prediction modelling for incoming call volume but have not yet advanced to predicting the nature of the incident (Brooks et al., 2011). Expanding on the principle of victim choice, an algorithm could be developed predicting the likelihood and timeliness of a response, given current incoming demand, and call complexity, providing an estimated time of arrival to be made. If police forces were able to do this, it would inform control room supervisors of potential delays enabling them to factor this into decision- making regarding triage. This information could be passed to victims when they call for service. Victims would then be aware of likely response times, and could then make an informed choice, opting to use R-TREC or await a non-urgent physical attendance. This would help to set expectations of victims and ensure that they have adequate safety advice. That would also help them make plans for the duration of any anticipated delay. That benefit, in turn, would enable police forces to become more responsive to victim needs. It might also ensure that specialist personnel, such as domestic abuse or mental health trained officers, were available for immediate telephone or video response at the appropriate times of the day and days of the week.

Advances in technology must be considered when considering the future expansion of R-TREC. Telephone communication is established and commonplace. Rapid technological advances in mobile phone camera technology, as well as a shift in video-calling culture because of the COVID-19 pandemic, which mean that the obvious next step for R-TREC would be to progress into video on the model of the treatment tested in the Kent Police Rapid Video Response (RVR) study (Rothwell et al, 2022). While this has happened already in Kent, it is important for other police agencies to replicate and extend these experiments.

The ambition of providing an in-person response to all victims or witnesses who call for police assistance appears to be no longer achievable. The data from this study is evidence of the continuing and relentless demand that calls for service brings to bear on police forces. Police forces should consider ceasing to promise a service that they do not have the resources to deliver, and either (1) set realistic expectations around the likelihood and timeliness of an in-person response or (2) radically alter response service offerings. The new National Policing Digital Strategy is based upon establishing new methods of contact and encourages police forces to design effective new response services that can meet the needs of the future (Police Service Digital, 2020).

This study shows that R-TREC can be added to the armoury of response services available to Police Chiefs, with the ability to service those calls that are lower in risk and least likely to receive an in-person response. The immediate telephone response service is cheaper and faster. At least for the kinds of incidents included in the experiment, this FAST response provides an evidence-based solution to the dilemma of demand exceeding capacity.
